# The formation of unsaturated zones within cemented paste backfill mixtures—effects on the release of copper, nickel, and zinc

**DOI:** 10.1007/s11356-018-2222-9

**Published:** 2018-05-13

**Authors:** Roger Hamberg, Christian Maurice, Lena Alakangas

**Affiliations:** Luleå University of Technology, 971 87 Luleå, Sweden

**Keywords:** Tailing management, Cement, Trace metal leaching

## Abstract

Flooding of cemented paste backfill (CPB) filled mine workings is, commonly, a slow process and could lead to the formation of unsaturated zones within the CPB fillings. This facilitates the oxidation of sulfide minerals and thereby increases the risk of trace metal leaching. Pyrrhotitic tailings from a gold mine (cyanidation tailing (CT)), containing elevated concentrations of nickel (Ni), copper (Cu), and zinc (Zn), were mixed with cement and/or fly ash (1–3 wt%) to form CT-CPB mixtures. Pyrrhotite oxidation progressed more extensively during unsaturated conditions, where acidity resulted in dissolution of the Ni, Cu, and Zn associated with amorphous Fe precipitates and/or cementitious phases. The establishment of acidic, unsaturated conditions in CT-CBP:s with low fractions (1 wt%) of binders increased the Cu release (to be higher than that from CT), owing to the dissolution of Cu-associated amorphous Fe precipitates. In CT-CPB:s with relatively high proportions of binder, acidity from pyrrhotite oxidation was buffered to a greater extent. At this stage, Zn leaching increased due the occurrence of fly ash-specific Zn species soluble in alkaline conditions. Irrespective of binder proportion and water saturation level, the Ni and Zn release were lower, compared to that in CT. Fractions of Ni, Zn, and Cu associated with acid-soluble phases or amorphous Fe precipitates, susceptible to remobilization under acidic conditions, increased in tandem with binder fractions. Pyrrhotite oxidation occurred irrespective of the water saturation level in the CPB mixtures. That, in turn, poses an environmental risk, whereas a substantial proportion of Ni, Cu, and Zn was associated with acid-soluble phases.

## Introduction

Cyanide is typically used to extract gold occurring as inclusions in sulfide minerals, such as pyrrhotite and pyrite. In the cyanide leaching process, lime addition can avoid the formation of toxic cyanide species by reaching a pH of ≈ 10. In refractory gold ores, an oxidation step aimed at oxidizing the sulfide minerals enclosing the gold is usually performed prior to the cyanide leaching process (Mesa Espitia and Lapidus 2015). The goal of this step is to increase the availability of cyanide for dissolving the gold. However, this process is seldom 100% effective, and remnants of sulfide minerals often occur in tailings obtained through a cyanide leaching process (cyanidation tailing (CT)). These sulfides often contain trace metals such as copper (Cu), arsenic (As), and zinc (Zn). Furthermore, sulfide minerals exposed to atmospheric oxygen and water may become oxidized. These reactions (reaction 1) yield sulfate and an acid leachate, referred to as acid mine drainage (AMD), with a high content of metals (Blowes et al. 2003). Owing to the co-occurrence of these trace metals and the sulfide minerals, CT must be carefully handled to avoid pollution of the surrounding environment.

1 $$ {\mathrm{FeS}}_2+15/4{\mathrm{O}}_2+7/2{\mathrm{H}}_2\mathrm{O}\to \mathrm{Fe}{\left(\mathrm{OH}\right)}_3+2{{\mathrm{SO}}_4}^{2-}+4{\mathrm{H}}^{+} $$

Tailing management often aims to restrict the interaction of tailings with the environment. A commonly used method is by placing the tailings in underground engineered facilities. Managing of CT in underground workings often includes the use of a so-called “Cemented paste backfill” (CPB) method. In CPB, low fractions (3–7 wt%) of cementitious binders are mixed with tailings and backfilled into underground facilities (Coussy et al. 2011). Using CPB can primary lead to the formation of a monolith that can serve as a geotechnical support to underground mine cavities, thereby increasing the operational benefits of the mining industry. Increasing the fraction of binders in a CT-CPB may yield intensified hydration and pozzolanic reactions, thereby enhancing the mechanical strength. The use of CPB could also prevent air intrusion into CT and thereby lower the sulfide-oxidation rate. Trace metal immobilization in CPB has been applied to a combination of physical encapsulation and chemical stabilization. Studies of Chen et al. (2009) and Paria and Yuet (2006) suggested that nickel (Ni) and chromium (Cr) can be encapsulated in the calcium-silicate-hydrate (C-S-H) structure. This physical encapsulation is largely dependent on the inherent strength of the CPB. The CPB strength results primarily from the abundance of C-S-H (Peyronnard and Benzaazoua 2012) formed by the addition of calcium-rich binders that hydrate in solution. In CT-based CPB materials (CT-CPB), dissolution of C-S-H contributes to neutralization of the acid formed by the oxidation of sulfides (reactions 1 and 2). Sulfide oxidation, gypsum dissolution, the cyanide leaching process, and/or the binders may generate sulfates in the CT and the CT-CPB mixtures. The stability of C-S-H in a CPB is governed by the sulfide and sulfate content, curing time as well as the type and fraction of binder material (Ercikdi et al. 2009; Kesimal et al. 2005; Benzaazoua et al. 2004). The cementitious phase reacts with sulfates, thereby forming expansive phases such as gypsum and ettringite (a sulfate attack, reactions 2 and 3) that may lead to a decrease in the inherent strength, generate cracks, and increase the availability of oxygen in the CPB material. A chemical stabilization may occur and the hydroxyl anions from this process may react with trace metals (i.e., Pb and Zn), forming hydroxides that can adsorb onto C-S-H. However, highly alkaline conditions (resulting from the release of OH^−^) could also increase the mobility of some trace metals (Cr, Pb, Zn, and Cu) (Kumpiene et al. 2008). Maintaining the stability of C-S-H is therefore essential from the viewpoint of trace metal mobility in a CT-CPB.

2 $$ \mathrm{Ca}{\left(\mathrm{OH}\right)}_2+2{{\mathrm{SO}}_4}^{2\hbox{-} }+2{\mathrm{H}}_2\mathrm{O}\to {\mathrm{CaSO}}_4\cdot 2{\mathrm{H}}_2\mathrm{O}+2{\mathrm{OH}}^{\hbox{-} } $$

3 $$ {\mathrm{Ca}}_4{\mathrm{Al}}_2{\left(\mathrm{SO}\right)}_4\cdot 12{\mathrm{H}}_2\mathrm{O}+2{{\mathrm{SO}}_4}^{2-}+2{\mathrm{Ca}}^{2+}+20{\mathrm{H}}_2\mathrm{O}\to {\mathrm{Ca}}_6{\mathrm{Al}}_2{\left({\mathrm{SO}}_4\right)}_3{\left(\mathrm{OH}\right)}_{12}\cdot 26{\mathrm{H}}_2\mathrm{O} $$

Maintaining a high grade of water saturation in a CT-CPB will hinder sulfide oxidation and the subsequent sulfate attack on the C-S-H. In field conditions, a CT-CPB is typically flooded when mine operations are disclosed and the ground water has recovered to natural levels. Complete flooding of a CT-CPB-filled stope may take several years, and unsaturated zones may form within the CT-CPB (Ouellet et al. 2006). This will increase the risk of a significant sulfide oxidation to occur within the CT-CPB. A previous study of Kesimal et al. (2005) showed that the strength of a CPB material (especially sulfide-rich CT-CPB) could decrease by more than 50% during prolonged (> 1 year) curing. Cruz et al. (2001) have shown that low fractions of binders may be insufficient to prevent the occurrence of AMD. Knowledge of trace metal and sulfide mineral mobility in CT-CPB is therefore essential for the prediction/management of contaminant release over an extended period of time. In some underground workings, the strength of the CT-CPB material is of less concern, but aims to minimize water percolation and enhancing trace metal stability still exist. So, when less strength is needed in the CPB material, how does it affect the leaching of trace metals? CT from a gold mine in the north of Sweden was to be managed by the use of a CPB application, whereas the strength demand was set to 0.2 MPa by the mine operator. The overall aim of this study was to determine whether a low-strength CPB (0.2 MPa) could be an environmentally friendly option for managing tailings (obtained through a cyanide leaching process) with elevated concentrations of Cu, Ni, and Zn.

As part of the overall aim, primary objectives of the study were to

- Evaluate the effect of using low-strength CT-CPB mixtures on the release of Ni, Cu, and Zn,
- Determine the effect of unsaturated conditions in low-strength CT-CPB mixtures on leaching of these elements.

The results of this study may increase knowledge of the preparation/management of CPB mixtures for use in mine facilities with slow recovery of natural groundwater levels.

## Materials and methods

Tailings and ore were collected from a gold mine in the north of Sweden. The ore was considered refractory as the gold occurred as inclusions in co-occurring sulfide minerals. According to personnel at the site, hydrogen peroxide (H_2_O_2_) was added in the 1st step of the cyanide leaching process, with the aim of oxidizing the sulfide minerals. This oxidation will increase the availability of cyanide for gold dissolution. Effluents from the H_2_O_2_ step were treated with lime (to maintain a pH of 10) to avoid the formation of hazardous cyanide species. Subsequently, Fe(SO_4_)_3_ was added for immobilization of the metal(oid)s. Fe(SO_4_)_3_ addition continued until a pH of ~ 8 was reached for the outlet water. Portland cement (CE) and biofuel fly ash (FA) were tested as cementitious binders for the preparation of various cemented paste mixtures. The ash was provided from a biofuel incineration plant located near to the mine site and could be classified as a class C fly ash (SiO_2_ + Al_2_O_3_ + Fe_2_O_3_ ≥ 60 weight (wt%)), according to ASTM C618-05 (2005). The main elements and total content of Cu, Ni, S, and Zn in the CT, ore, CE, and FA are presented in Table 1.

**Table 1:** Total content of elements and pH in the ore, tailings, fly ash, and cement (a selection from 31 elements is presented) (*n* = 3, ± SD)

	Ore	CT	Fly ash	Cement
Total solids (TS)	99.97 ± 0.06	89.0 ± 0.4	95.2 ± 0.7	99.4 ± 0.0
SiO_2_	30.3 ± 0.9	55.0 ± 4.9	34.6 ± 1.3	20.6 ± 0.8
Al_2_O_3_	1.74 ± 0.07	4.69 ± 0.04	10.7 ± 0.6	5.61 ± 0.45
CaO	4.26 ± 0.09	4.83 ± 0.25	14.1 ± 1.0	50.3 ± 1.8
Fe_2_O_3_	12.6 ± 0.5	16.7 ± 0.6	13.9 ± 1.0	2.81 ± 0.05
K_2_O	0.32 ± 0.00	0.92 ± 0.03	2.89 ± 0.08	0.83 ± 0.05
MgO	2.38 ± 0.07	3.24 ± 0.01	2.54 ± 0.08	4.00 ± 0.17
Cu	82.0 ± 12.1	147 ± 7	136 ± 10	86.2 ± 2.9
Ni	126 ± 12	63.8 ± 2.1	114 ± 9	63.8 ± 1.3
S	25,367 ± 2363	20,933 ± 493	13,700 ± 200	9960 ± 219
Zn	10.6 ± 1.5	25.0 ± 0.4	374 ± 10	149 ± 3
Paste-pH	N.D	5.03 ± 0.25	13.2 ± 0.6	12.7 ± 0.3

*N.D* not determined

In Hamberg et al. (2016), X-ray diffraction (XRD) and scanning electron microscopy combined with energy dispersive spectroscopy (SEM-EDS) revealed that pyrrhotite and arsenopyrite were the main Fe- and As-sulfide minerals in the CT. Quartz constituted the main mineral (~ 80 wt%) in the studied tailings. Tremolite, albite, and microcline each accounted for < 5 wt% of the tailings, and no Cu-, Ni-, or Zn-carrying minerals were found. The acid-base accounting (ABA) test was conducted in Hamberg et al. (2015) and showed that the tailings were considered to be acid generating with an acid potential (AP) of 65.3, neutralization potential (NP) of ~ 4.5. This rendered a net neutralization potential (NNP) of − 60.5. Calculation performed in Hamberg et al. (2016) suggested that the sulfide and sulfate content in CT accounted for 44 and 56 wt%. These estimations were based on the suggestions of Dold (2003), which encountered the sulfate content to be the amount of S released in steps 1–4 during the sequential extraction test. The sulfide content was equal to total S released in step 5–6.

### Sample analysis

The pH of the water samples was measured on unfiltered samples. The pH of the water samples was measured on unfiltered samples. The paste-pH was measured by a methods suggested by Sobek et al. (1978) and Page et al. (1982). Twenty grams of solids were mixed with 20 g of deionized water, stirred for 5 s, and then left for 10 min before pH was measured. A pH meter called “Meterlab PHM201” pH-Eh meter calibrated against standard buffers at pH 4.01 and 7.0 was used. Element compositions in the water samples were analyzed by a laboratory accredited by the Swedish board of accreditation and conformity assessment (SWEDAC). Total contents of Cu, Ni, and Zn were determined by high-resolution inductively coupled plasma-mass spectrometry (HR-ICP-MS), whereas Al, Ca, Fe, K, Mg, Na, and S were determined by inductively coupled plasma atomic emission spectroscopy (ICP-AES). Prior to analysis, samples were acidified with super-pure nitric acid using 1 mL of acid/100 mL of sample. Analyses of HR-ICP-MS and ICP-AES were performed using modified versions of the US Environmental Protection Agency (US EPA) methods 200.8 and 200.7, respectively.

Solid samples were analyzed for major and minor elements by the accredited laboratory. For the determination of Cu, Ni, S, and Zn, samples were dried at 50 °C, digested with 7 M nitric acid in closed Teflon vessels, and treated in a microwave oven. Concentrations of other elements were determined after fusion with lithium metaborate, followed by dissolution in dilute nitric acid. The solutions were subjected to centrifugation and diluted before analysis. For quality control, two in-house reference materials were analyzed in parallel with the solid samples. The dry matter content of CT, CE, and FA samples were determined in triplicate by drying the samples in an oven at 105 °C for 24 h according to Swedish standard SS 028113-1 (SIS 1981).

### Preparation of CT-CPB mixtures

Mixtures (desired strength 0.2 MPa) were designed with minimal fractions of binders (see Table 2). The tailings were mixed with the binders (CE and FA), and ~ 25% wt% of distilled water was slowly added (see Table 2 for the composition of the CT-CPB mixtures (CE and CE-FA)). The preparation is described in detail by Hamberg et al. (2015). CT-CPB mixtures of CE and CE-FA were cured for 31 or 446 days (referred to as CE31, CE446, CE-FA31, and CE-FA446 hereafter). CE31 and CE-FA31 were kept at room temperature at ~ 80% humidity under dark conditions, until the 31st day. In addition, CE446 and CE-FA446 were cured under the same conditions as CE31 and CE-FA31 until the 31st day, and subsequently at ~ 60% humidity, in the dark, under room temperature conditions until the 446th day. These conditions were chosen to enhance the formation of an unsaturated region at the surface of the CPB mixtures, as described by Ouellet et al. (2006). The curing period was terminated when brighter (drier) regions (compared with those in the bulk) formed on the surfaces of the CPB mixtures.

**Table 2:** Composition weight % (wt%) of cemented paste backfill (CPB) mixtures containing cement (CE) and/or fly ash (FA) and unmodified cyanidation tailings (CT)

Names	Portland cement (wt%)	Biofuel fly ash (wt%)	Total binder/dry weight (wt%)	Water content (wt%)	Tailings (wt%)
CE	100		1	26	73
CE-FA	67	33	3	26	71
CT	0	0	0	11	89

### Leaching tests

#### Flooded monoliths—tank leaching test

Tank leaching tests (TLTs) were conducted, in accordance with Dutch standard EA NEN 7375: 2004, on CT, CE-FA31, CE-FA446, CE31, and CE446. Duplicate samples were removed from the bottles after 31 or 446 days and shaped into regular cylinders. The overall experimental setup is described in Hamberg et al. (2015). CE 446 fell apart upon contact with water and, therefore, placed in paper filter bags (with 0.45-μm pores) inside nylon sample holders. The filter bags containing the granular CT and CE mixtures were changed upon each leachate renewal and were fully immersed in the water. In addition, the water was exchanged and analyzed after 0.25, 1, 2.25, 4, 9, 16, 36, and 64 days. The mass transfer of Cu, Fe, S, Ca, Zn, and Ni from CT and the CT-CPB materials was calculated from (as specified in the EA NEN 7375: 2004 standard):

4 $$ {M}_{\mathrm{ti}}=\left({C}_{\mathrm{i}}\times {V}_{\mathrm{i}}\right)/A $$

Where, *M*_ti_ (mg/m^2^): mass of the element released during leaching period i; *C*_i_ (mg/L): element concentration for period I; *V*_i_ (L): leachate volume for period I; and *A*: specimen surface area (m^2^) exposed to the leachate. The apparent diffusion coefficient of Cu, Fe, S, Ca, Zn, and Ni was determined by plotting the logarithm of the cumulative release Mt until the ith period against the logarithm of the time elapsed since the start of the experiment. A plot with a slope (*r*_c_) of < 0.4 is expected for the surface wash-off process, where surface-captured elements are released from the surface of the CT-CPB. The slope is defined as the logarithm of the total mass of the transferred elements plotted against the logarithm of the time elapsed during the experiment. Conversely, diffusion-controlled release should yield a linear plot with a slope of 0.4–0.6, and dissolution controls the release if the slope is > 0.6.

#### Sequential extraction test

Fractionation of Ca, Fe, S, Ni, Zn, and Cu was assessed using a modified sequential extraction scheme described by Dold (2003). In each extraction sequence, 2 g of CT, ore, CE446, and CE-FA446 were used and extracted with five different solutions consecutively. Materials from CE446 and CE-FA446 were taken from the outermost (brighter) regions with a lower water saturation level than the other regions. Overall details about the extraction procedure are presented in Hamberg et al. (2016).

#### Weathering cell test

Approximately 70 g of tailings was placed on a paper filter in a Büchner-type funnel. Duplicate samples were exposed to 31 weekly cycles (217 days), involving 1 day of leaching, 3 days of ambient air exposure, a second day of leaching, and two subsequent days of air exposure. The samples were leached by covering each with 50 mL of Milli-Q H_2_O for ~ 2 h. The leachates were then recovered by applying a vacuum to the funnel. The pH, Eh, and EC of the filtered leachates were subsequently measured; the chemical composition of the water samples was determined as described in Hamberg et al. (2016).

#### pH-dependent leaching test

The pH-dependent leaching test was inspired by the previous study of Peyronnard et al. (2009). Surficial materials of CE-FA446 and CE446 were crushed into a pulverized material, divided into 12.5-g sub-samples, and placed in acid-cleaned plastic bottles. The aim of the test was to estimate the release of Cu, Ni, and Zn in response to a decrease in the pH of the CPB material. A more detailed description of the test is described in Hamberg et al. (2017). The pH ranged from 8 to ~ 2.7 corresponding to the normal pH of CE446, CE-FA446, and unmodified tailings, respectively. The bottles were stirred for 8 days to reach a constant pH, and then leachates were filtered with 0.45-μm polypropylene membranes on a Büchner filter.

#### Modeling—PHREEQC

Speciation-solubility calculations were performed with the geochemical code PHREEQC (Parkhurst and Appelo 1999) using the ThermoDem database (Blanc et al. (2007), version V1:10, which includes cementitious phases inferred by Lothenbach et al. (2008). Element concentrations, redox potential (*E*_h_), and pH in leachates from the TLT were used in these calculations.

### Scanning electron microscopy

A JSM-IT 100 scanning electron microscopy (SEM), by JEOL, was used for the observation of particle morphology. Investigated samples were dried, powdered, and placed over a conductive carbon tape. Secondary electron detector (SED) mode, with high vacuum and acceleration of 10 kV, is used to obtain all the micrographs. The morphology is analyzed at different magnifications.

## Results

### Sequential extraction test

**Fig. 1:**
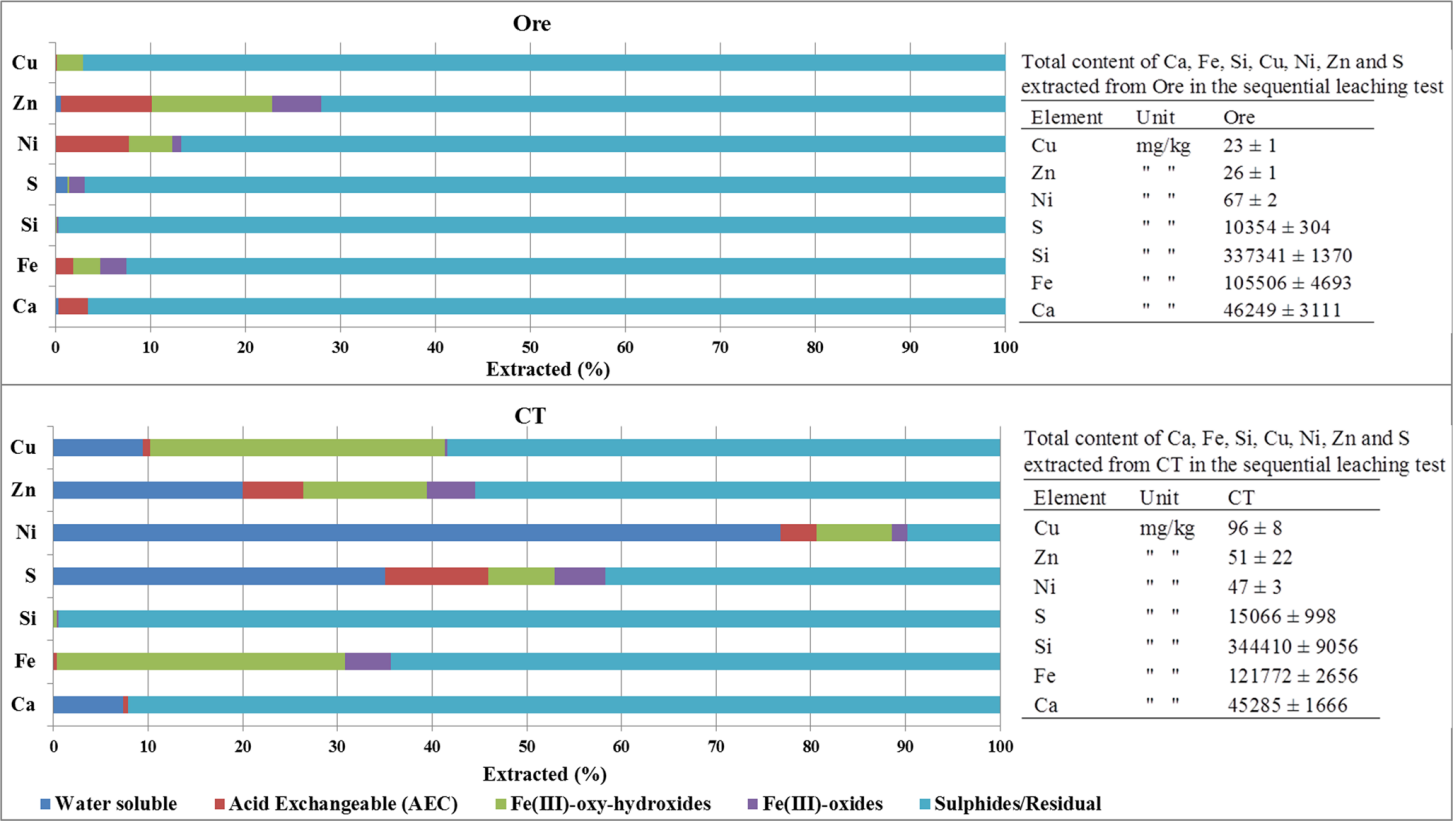
Fractionation of Ni, Zn, Cu, Fe, S, Si, and Ca in the ore and CT (average values presented, *n* = 3). Total content (*n* = 3; ± SD) of extracted elements in attached tables

Sequential extractions were conducted on triplicate samples of ore, CT, CE446, and CE-FA446. A majority of all the analyzed elements in the ore was associated with the sulfide/residual fraction. Less than 30 wt% of Zn was associated with more soluble phases. The corresponding proportions of Cu, Ni, S, Ca, Fe, and Si associated with more soluble phases were 3–12 wt%. In the ore, there were no or very small amounts of analyzed elements associated with the water soluble phase. In CT, proportions of elements associated with the sulfide/residual fraction were lower compared to that in the ore, especially that of Ni. Instead, significant proportions of Ni (77 wt%), Cu (~ 10 wt%), Zn (~ 20 wt%), Ca (~ 8 wt%), and S (35 wt%) were associated with the water soluble fraction in CT. Concerning Fe and Si, minor amounts in CT were associated with the water-soluble phase. The proportions of Cu, S, and Fe associated with AEC, Fe(III)-oxy-hydroxide, and Fe(III) oxides in CT were higher compared to that in the ore, but unchanged for Zn and Si (Fig. 1). Total extracted amount of Cu was significantly higher in CT compared to that in the ore (Fig. 1). Detailed results from the sequential extraction tests are attached in an appendix Table 4.

**Fig. 2:**
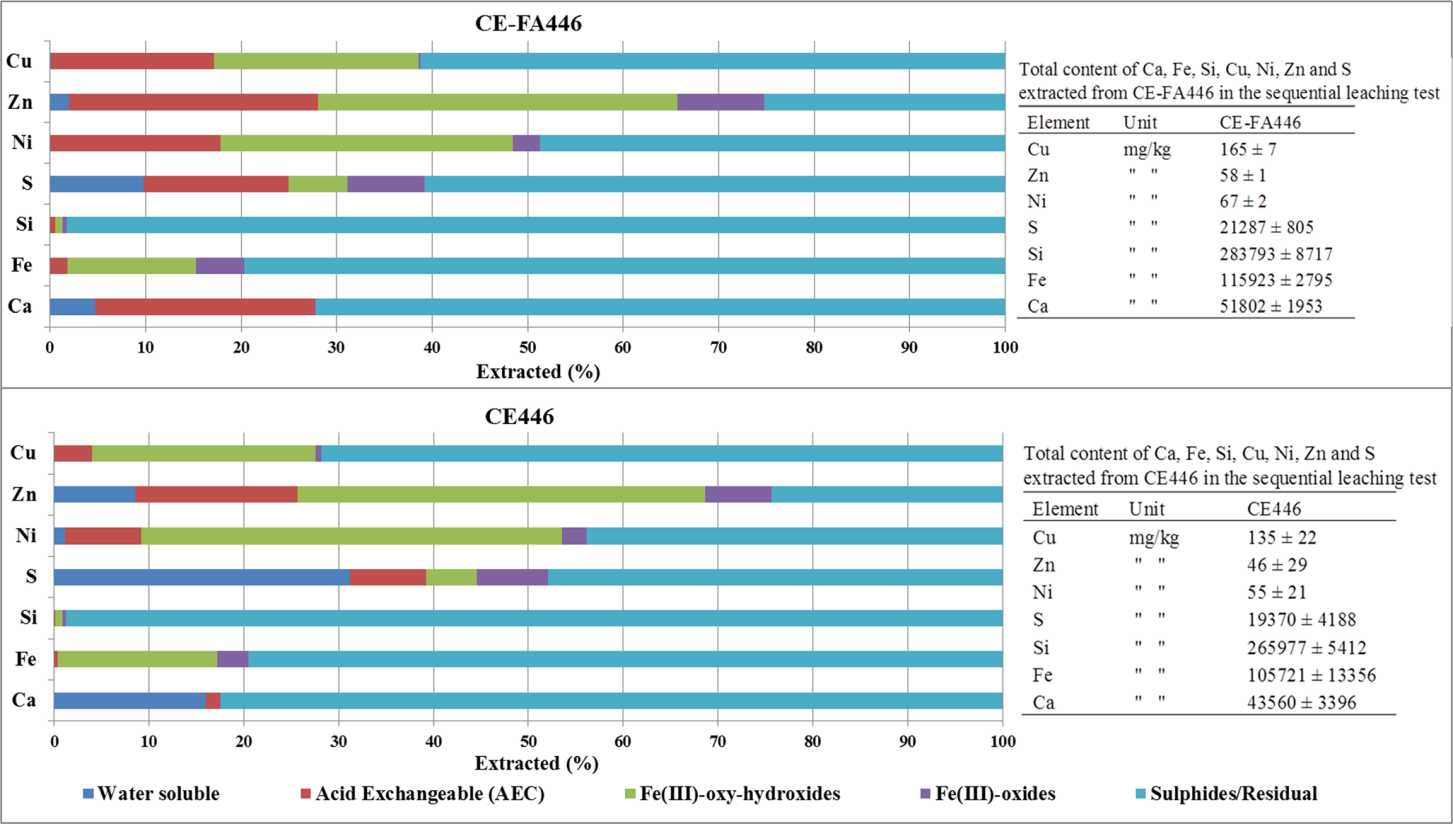
Fractionation of Ni, Zn, Cu, Fe, S, and Ca in the CE446 and CE-FA446 (average values presented, *n* = 3). Total content (*n* = 3; ± SD) of extracted elements in attached tables

In the CT-CPB mixtures, less proportions of Cu, Ni, and Zn were associated with the water-soluble fraction, compared to that in CT. The opposite concerned for S in CE-FA446. In general, higher proportions of Ni, Zn, Cu, Fe, S, Si, and Ca in CE-FA446 were associated with the AEC fraction, compared to that in CE446. This difference is most significant concerning Ca (Fig. 2). Proportions of Ni, Zn, Cu, Fe, S, Si, and Ca associated with Fe(III)-oxy-hydroxide and Fe(III) oxides were similarin CE-FA446 and CE446 (Fig. 2). Detailed results from the sequential extraction tests are attached in an appendix Table 4.

Compared with CT, CE and FA contained higher total concentrations of Ni and Zn. Similarly, Ni and Zn occurred with higher fractions in the CT-CPB mixtures (CE31, CE-FA 31, CE-FA446, and CE446) than in the CT. An addition of CE increased the fraction of Ni by 1.8%, in the CE mixture, compared to that in CT. The corresponding increase in CE-FA was 21.7 (Zn) and 3.0% (Ni).

### Tank leaching test

**Fig. 3:**
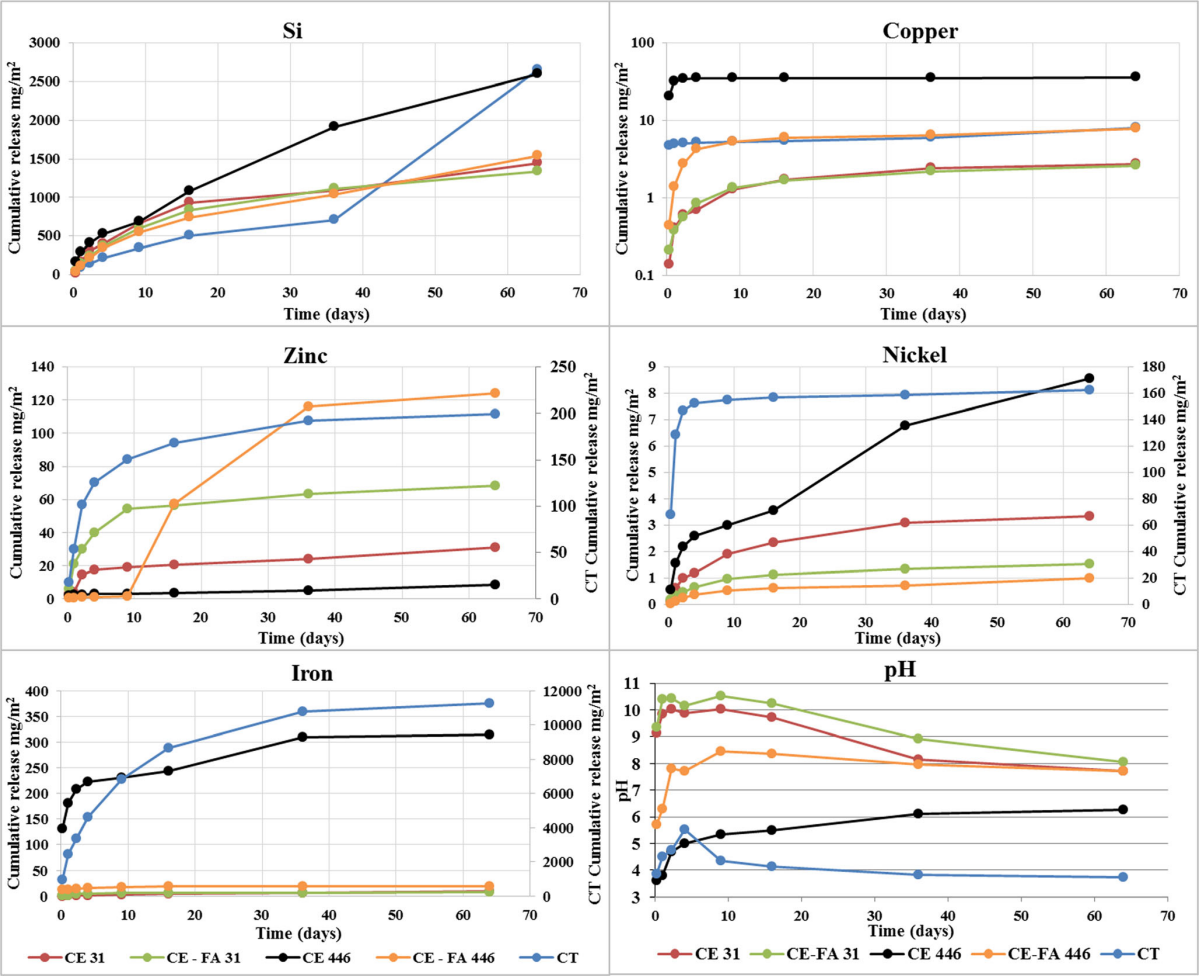
Evolution of pH and cumulative release of Si, Cu, Zn, Ni, and Fe (in mg/m^2^) from CT, CE-FA31, CE31, CE-FA446, and CE446 during TLT (averaged values, *n* = 2)

In the TLT, small deviations are observed in results from the duplicated tests of CT, CE 31, CE 446, CE-FA 31, and CE-FA 446 and, therefore, average values are presented. The release of Fe, Ni, Zn, and Si from CT was significantly higher than that released from the CT-CPB-materials. The opposite concerned for Cu, whereas higher amounts were released from CE446. In the CT-CPB materials, the largest amount of Fe, Ni, and Si was released from CE446 (Fig. 3), while the most abundant release of Zn was encountered from CE-FA446. In all materials, the release of Cu and Fe evolved in a similar way and increased considerable during the first days (until the ninth day) of the TLT but was lowered toward the end. This was also somewhat true for the evolution of Ni and Zn. However, the Zn release in CE-FA446 increased steeply during day 9–36 and was significantly lower in the last extraction. A Ni release from CE446 increased throughout the TLT (Fig. 3). The release of Si was most abundant in CT and increased steeply toward the end of the TLT. At the last extraction, the Si release from CE31, CE-FA446, and CT increased, whereas the opposite occurred in CE-FA31. A Si release from CE446 increased throughout the TLT. The pH evolved in a similar way in CE31 and CE-FA31. In each case, the pH increased initially, stabilized at ~ 10–10.5, and dropped to 8 during the last extractions. The pH of CE446 and CE-FA446 were lower than those of CE31 and CE-FA31. Furthermore, the pH of CE-FA446 increased initially and stabilized at a pH of 8. In CE446, pH increased throughout the TLT from 3 to ~ 6.

**Fig. 4:**
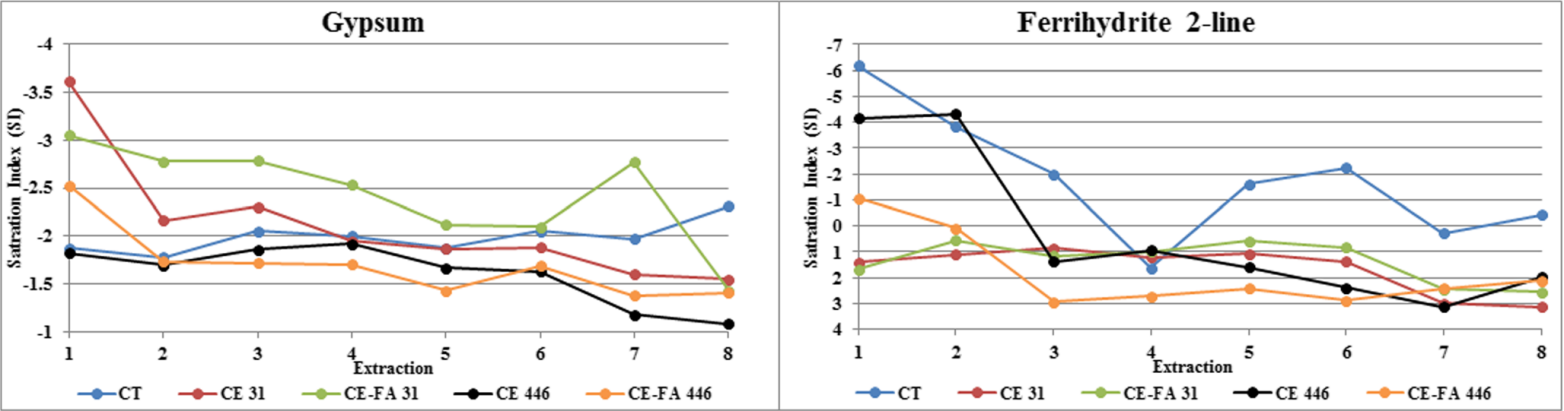
Saturation indexes of gypsum and two line (2 L) ferrihydrite during TLT

The saturation index of gypsum in CT decreased during the TLT. The saturation indexes of ferrihydrite in CE 446 and CE-FA 446 were negative (suggesting dissolution) during extractions 1–2, but positive during extractions 3–8. Furthermore, the saturation indexes of ferrihydrite in CT were negative during all extractions, except for extraction 4 (Fig. 4).

Results of the calculations based on the EA NEN 7375: 2004 standard indicated that similar Cu and Ni leaching mechanisms operate in CE31 and CE-FA31 (Table 3). In the CT-CPB materials, the cumulative release of Zn from CE-FA31 and CE-FA446 was higher than that from CE31 and CE446. Similarly, more Fe and Cu were released from CE-FA446 and CE446 than from CE31 and CE-FA31. The release of Cu and Ni from CE31 and CE-FA31 occurred via diffusion induced by the dispersal of these elements from pore water. However, the release of Zn from the granular materials (CT and CE446) and the monoliths (CE-FA446, CE31, and CE-FA31) was induced by wash-off behavior and diffusion, respectively (Table 3).

**Table 3:** Cumulative amounts of elements (in mg/m^2^) released via the dominant leaching mechanism during TLT

Mixture	Cu	Ni	Zn
CT	8.0 DF	162 SW	199 SW
CE 446	35 SW	8.6 SW	8.9 SW
CE 31	2.7 DF	3.3 DF	31 DF
CE-FA 446	7.7 DF	1.0 DF	124DS
CE-FA 31	2.5 DF	1.5 DF	68 DF

*DF* diffusion, *SW* surface wash off, *DS* dissolution

### Weathering cell test

After 217 days of the weathering cell tests, the cumulative release of Ni, Zn, Fe, and Cu from the CT-CPBs was lower than that from CT. More Cu was released from CE-FA than from CE-FA, but the opposite was true for Ni and Zn. Similar pH trends were observed for CE and CE-FA. In both cases, the pH of the washings decreased from 11.4 (CE-FA) or 10.1 (CE) to a stable value of ~ 8 after 10 days (Fig. 4). Small deviations were observed in the results of the duplicated tests of CT, CE, and CE-FA, and, therefore, average values are presented. The release of Ni from the CT-CPB samples seemed to be correlated with changes in the pH (i.e., the maximum release occurred when the pH decreased from 11 to 8). Similar Zn and Cu evolution was observed in the CT-CPB samples, with maximum values occurring on the 10th day of the weathering cell test (WCT). The values then decreased until day 24, but then increased until day 52. The amount of Si decreased until the tenth day of the experiment, but increased thereafter. Prior to the addition of acid (after day 70), a low amount of Fe was released from the CT-CPBs.

**Fig. 5:**
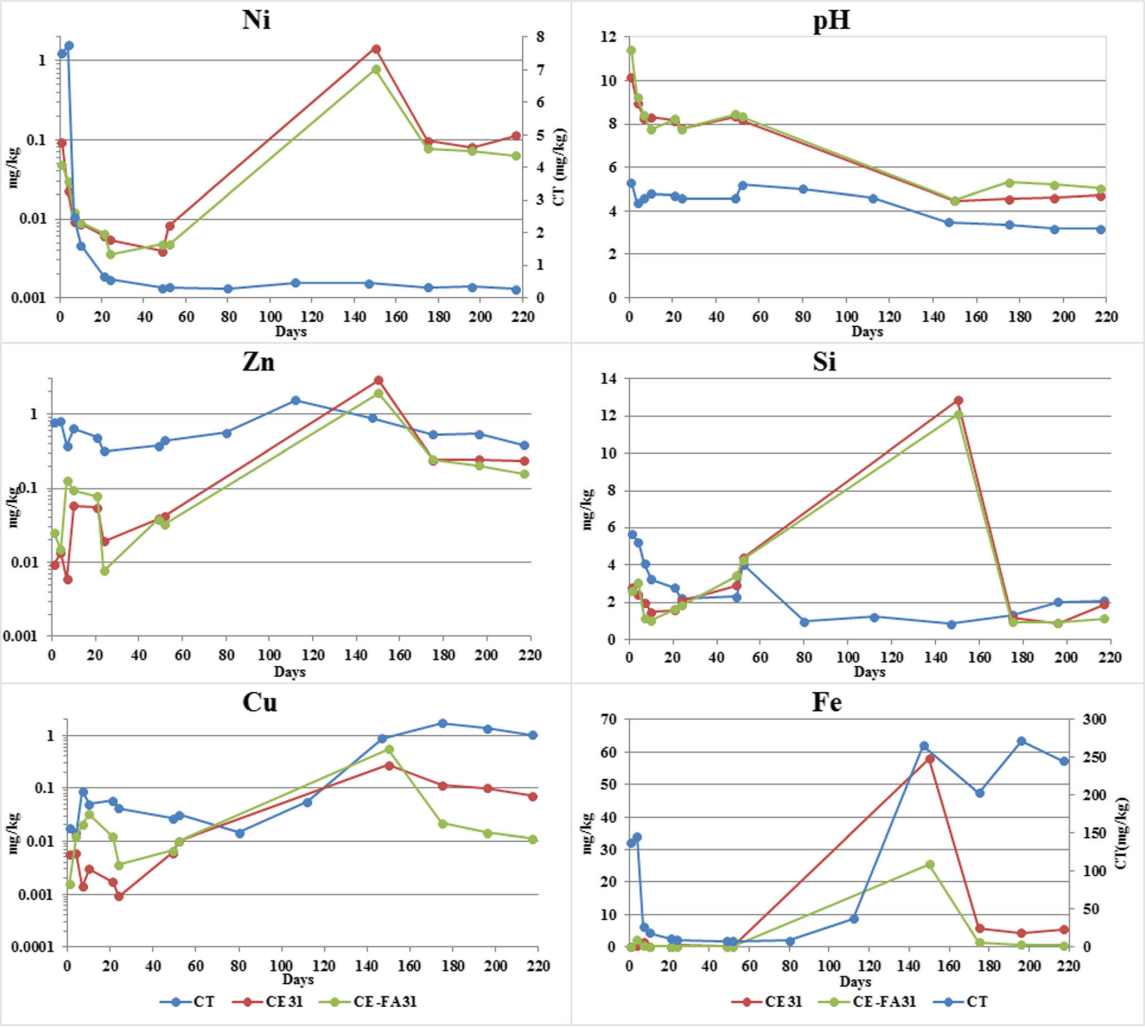
Evolution of pH and release of Ni, Zn, Cu, Si, and Fe from CT, CE31, and CE-FA31 with time during the WCT. Acid was added during days 70–147; no acid was added to CT

After 70 days, acid was added to the leachate with the aim of consuming the buffering minerals in the CPB materials. Different amounts of acid (1 M HCl) were added over 77 days (between days 70 and 147) to each CPB material (CE 0.47 M H^+^/kg TS and CE-FA 0.69 M H^+^/kg TS). Consequently, the pH of each material decreased to ~ 4.5, i.e., the initial value determined for the unmodified tailings. Small deviations were observed in the results of the duplicated tests of tailings CE and CE-FA, and, therefore, average values are presented. The leaching of Ni, Zn, Si, Cu, and Fe from CE and CE-FA increased with the addition of acid. At 217 days, the cumulative release of all the presented elements from the CT-CPBs was lower than the release from the CT. With the addition of acid, the leaching of Si, Fe, Zn, and Ni from CE was more strongly increased than the corresponding leaching from CE-FA. Endpoint pH values of (i) ~ 4.5 and (ii) 3.2 were obtained for (i) CE and CE-FA and (ii) the tailings, respectively. Cu and Fe leaching from the unmodified tailings increased during WCT, and pH values of < 3.5 were obtained (Fig. 5).

### pH-dependent leaching test

**Fig. 6:**
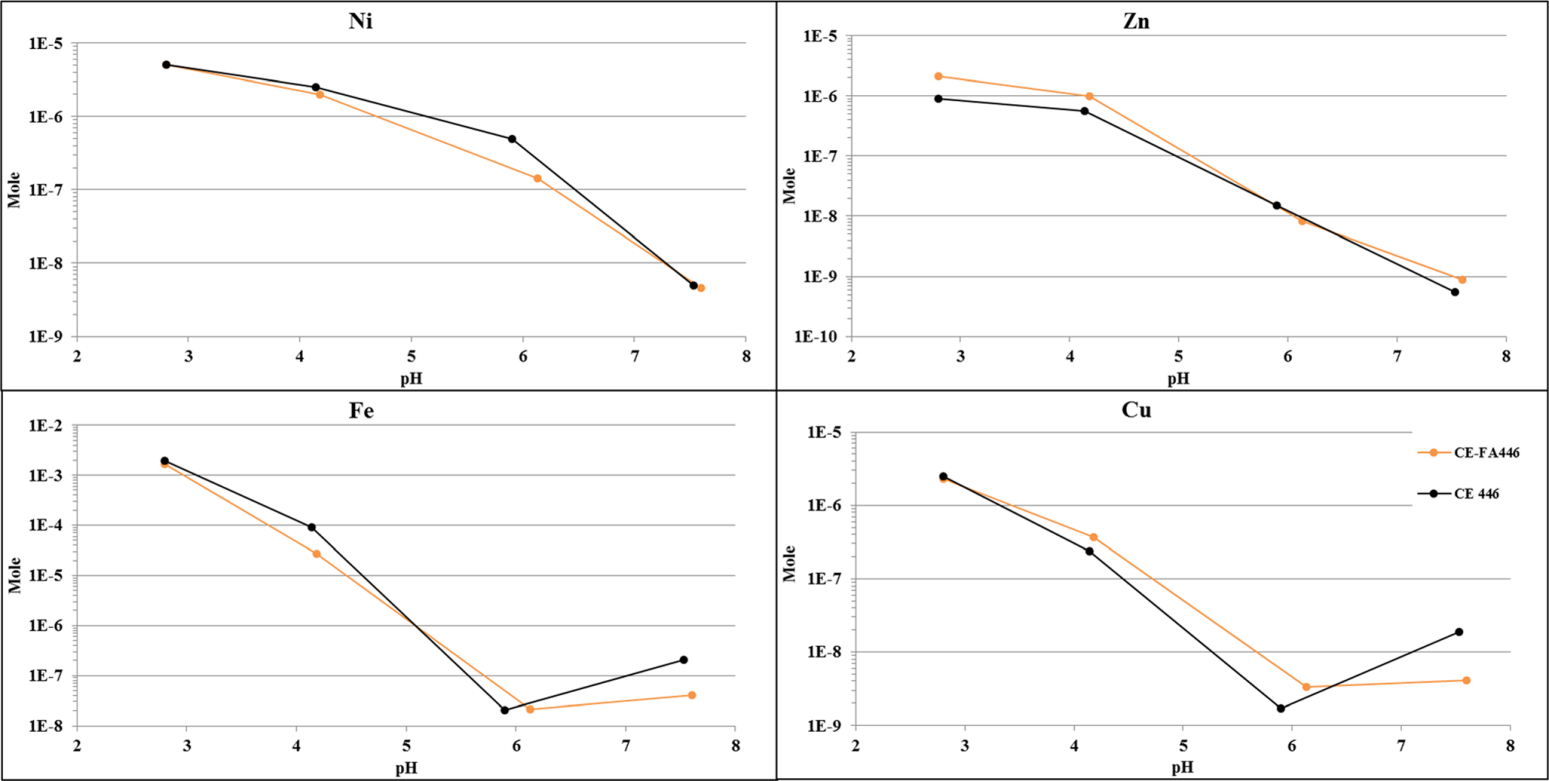
The release of Ni, Cu, Fe, and Zn (expressed in mole) during the pH-dependent leaching test (averaged release, *n* = 2)

The Ni and Zn release from CE446 and CE-FA446 increased in tandem with a pH decrease. The release of Cu and Fe experienced a different leaching pattern and was more abundant at pH 8 compared to that in pH 6 (Fig. 6).

### Scanning electron microscopy

**Fig. 7:**
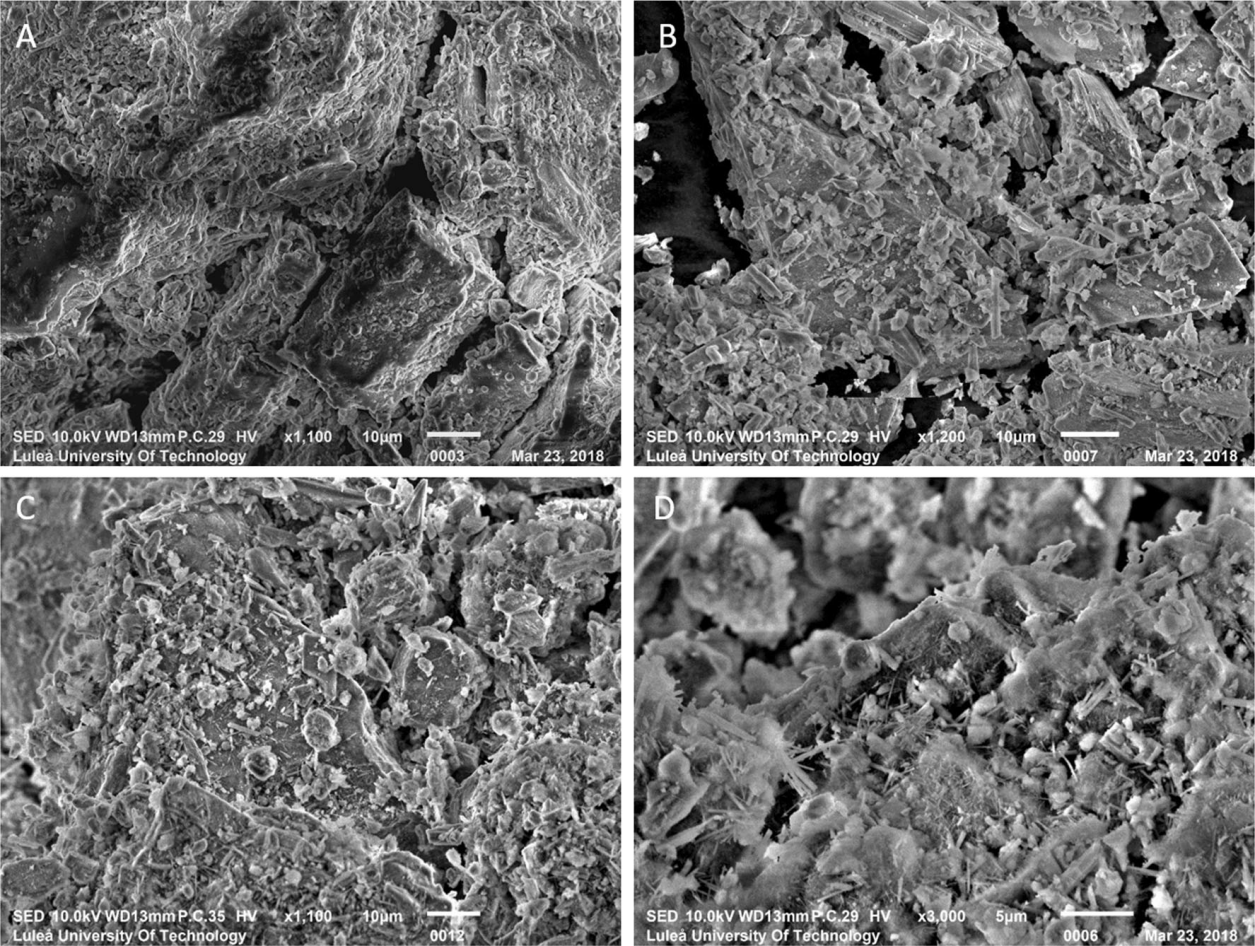
SEM micrographs of CT (**a**), CE446 (**b**), and CE-FA446 (**c**, **d**)

In Fig. 7a, tabular-shaped particles with approximately length of 30–40 μm suggested the presence of gypsum (Myagkaya et al. 2016). In addition, typical gypsum morphology particles are detected in CE-446, Fig. 7b. From Fig. 7c, d, it was visible that tailings treated with CE and FA have developed needle-shaped morphology, typical of the hydrated cement paste phases, suggesting the formation of C-S-H and ettringite (Panchal et al. 2018). In addition, typical spherical shaped fly ash particles are observed in CE-FA446, Fig. 7d (Grau et al. 2015).

## Discussion

### Unsaturated conditions in CT-CPB mixtures—effect on the solubility of cementitious binders

Immobilization of trace metals in cementitious mixtures has been applied to physical encapsulation and chemical stabilization (Chen et al. 2009; Paria and Yuet 2006; Benzaazoua et al. 2004). The physical strength is strongly dependent on the stability of cementitious C-S-H. Ideally, cementation will reduce the open porosity and obstruct water percolation through the CT-CPB mixtures. In these mixtures, the addition of cementitious binders was probably manifested as an increase in the amount of Ca and S associated with the AEC fraction (Dold 2003). Pyrrhotite oxidation generated sulfates that dissolved the binders, resulting in the formation of gypsum and ettringite (reaction 4) and, in turn, a reduction in the mechanical strength. Dissolution of this kind is suggested by the presence of C-S-H and ettringite in CE-FA446 (Fig. 7). The solubility of the cementitious phases increased at pH values of < 9 (Benzaazoua et al. 1999). The evolution of pH in leachates from all the CT-CPB mixtures during the TLT suggested that the cementitious phases have dissolved to some extent, especially in CE446 that fell apart into a granular material. Cementitious phases were more abundant in CE-FA446 compared to that in CE446 that seems to contain more gypsum (Fig. 7). The low strength of CE446 was probably also reflected in the larger amount of gypsum formed, as indicated by the higher fraction of water-soluble Ca and S (Dold 2003), compared with that of CE-FA446 (see Fig. 2).

### Unsaturated conditions in CT-CPB mixtures—effect on the release of Ni, Cu, and Zn

According to Sciuba (2013), the major sulfide minerals in Svartliden consist of pyrrhotite (Fe_7_S_8_) and arsenopyrite (FeAsS). However, traces of chalcopyrite (CuFeS_2_), pyrite (FeS_2_), and sphalerite (ZnFeS) are also found. No chalcopyrite, sphalerite, and Ni species was found during mineralogical analyses, but it is a common fact that mining processes is never 100% effective, so remnants of sulfides are probably present in CT. Results from the fractionation (Fig. 1) suggested that significant amounts of sulfides in the ore oxidized during the cyanide leaching process. A substantial fraction of Zn, Cu, and Ni from this process was re-distributed to water-soluble-species and/or co-precipitated with Fe-(oxy)-hydroxides. The cyanide leaching process yielded a pH of ≈ 8 for the outlet water. This value resulted from the addition of lime and consequent formation of metal hydroxides (i.e., Cu(OH)_2_, Zn(OH)_2_), and Ni(OH)_2_) (Donahue et al. 2000; Hidmi and Edwards 1999). These hydroxides are stable under pH-neutral, oxidized conditions (Karapınar 2016; Kumpiene et al. 2008). In CT, pyrrhotite oxidation products leads to acidic conditions (pH: 3.5–5.0) in TLT-leachates (Fig. 3) that probably dissolved a majority of these hydroxides. A majority of the Zn, Ni, and Cu were rinsed of the surface via a wash-off-effect in CT (Table 3) whereas initially high amounts of these elements decreased toward the end TLT (Fig. 3). This evolution is typical for a wash-off-effect, probably induced by the formation of a reddish crust on the surface of CT mid-way through the TLT. The crust was assumed to be an enclosing iron-oxide precipitate. As hydroxides of Ni, Cu, and Zn dissolves, these could be re-adsorbed onto Fe precipitates, but this process becomes less effective in acidic conditions. In addition, Ni release and Zn release were more than ten times higher than the Cu release in the CT. This may have resulted from the fact that Cu is readily adsorbed onto Fe precipitates at a pH of 4, whereas other metals (i.e., Ni and Zn) remain in the solution (Dzombak and Morel 1990).

In CE31 and CE-FA31, the dissolution of the cementitious phases increased the pH of the TLT leachates to alkaline (pH 8–10.5) levels. The diffusion-like behavior of Cu, Zn, and Ni suggests that these elements were transported from the CT-CPB pore water to the surrounding leachate. Heavy metals in a CPB matrix could occur as metal hydrated phases or metal hydroxides precipitated on C-S-H-surfaces (Li et al. 2001). In these cases, a metal release occurs as the C-S-H:s are dissolving, causing alkaline conditions in the CT-CPB-leachates. This phenomenon could result in the formation of anionic complexes with a low affinity for Fe precipitates in the pore water of CE31 and CE-FA31. A Ni and Cu release could then be induced by desorption from Fe precipitate surfaces (Kumpiene et al. 2008). The Zn release from the CT-CPB mixtures was approximately a tenfold higher than the Ni and Cu release and most extensive at pH levels of < 8 or > 10 (Fig. 3). A Zn release was probably governed by zinc hydroxides (Zn(OH)_2_) that are amphoteric and mobile in both alkaline and acidic conditions (Malviya and Chaudhary 2006). Zinc hydroxides could gradually be transformed into cationic species (Zn^2+^, Zn(OH)^+^) as the pH decreased to < 10 and anionic species at pH > 10 (Degen and Kosec 2000). Anionic Zn species could then be released due to desorption from Fe precipitates. The Zn release may also origin from the fly ash that, compared to CT, contains substantially higher fractions of Zn (Table 1). In the CE-FA mixtures, fly ash could contain Zn attached to calcium alumino-silicate glass that is more soluble under alkaline than acidic conditions (Luo et al. 2011). That might explain a comparatively higher release of Zn in alkaline conditions. Overall, under saturated conditions, the amounts of Cu, Ni, and Zn released from the CT-CPBs were lower compared to that in CT. Furthermore, this release (from the CT-CPBs) was governed by the stability of the cementitious phases and/or desorption from Fe precipitates.

**Fig. 8:**
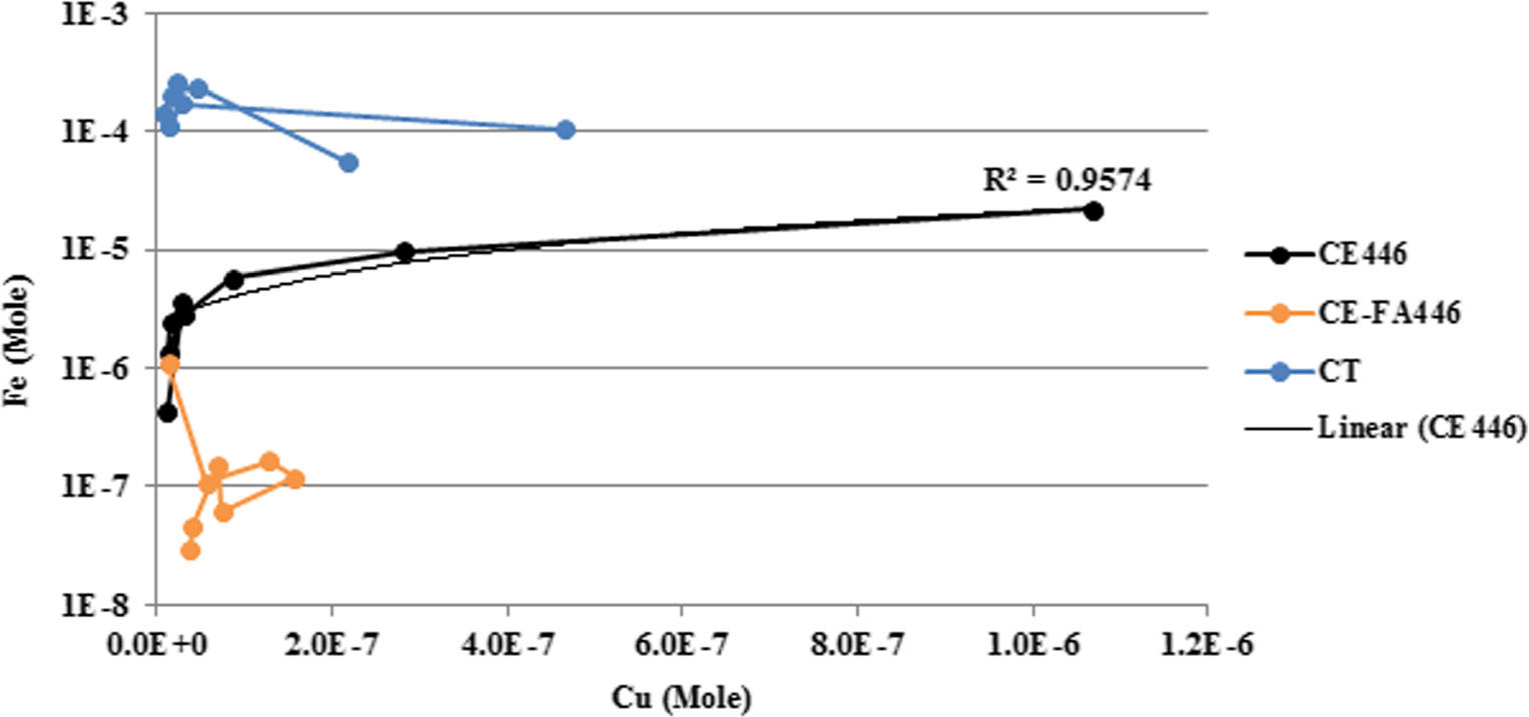
Release of Cu and Fe (expressed in mole) from CE446, CE-FA446, and CT during the TLT, linear regression analyses (*R*^2^)

The establishment of unsaturated conditions in the CT-CPB mixtures resulted in increased pyrrhotite oxidation and promoted acidic conditions in the TLT leachates. In the TLT leachates, initial pH was lower in CE446 compared to that in CE-FA446. In CE446, pyrrhotite oxidation may have progressed more efficiently compared to that in CE-FA446. Increasing the binder proportion by adding FA (such as in CE-FA mixtures) may enhance the microstructure and decrease the chemical (oxygen transport) reactivity in the CT-CPB mixtures, by reducing the diffusion rate of oxygen through the CT-CPB mixtures (Aldhafeeri and Fall 2017). A Cu release increased, especially in CE446, and was higher than the Cu release from CT. An increase in the Cu leaching was most evident at pH < 4 and was probably correlated with the dissolution of ferrihydrite (Fig. 4). According to Munk et al. (2002), Cu has a sorption edge onto ferrihydrite at a lower pH compared to that of Ni and Zn. The fraction of Cu released in neutral/alkaline conditions could therefore be higher; this is also seen in the pH-dependent leaching test (Fig. 5). The Cu fraction desorbed is probably secondarily connected to Fe(III) oxy-hydroxides, which are more easily released (especially in the acidic range) than the originally formed Cu-Fe precipitates (Munk et al. 2002). This probably resulted in a greater release of Fe-associated Cu (especially under acidic conditions) from CE446, during the TLT, than from CT (Fig. 8).

An establishment of unsaturated zones in the CT-CPBs had a negligible effect on the Ni release, which was low during the TLT. The Ni release from CE446 was more abundant compared to that in CE-FA446. This could be due to a more abundant dissolution of amorphous Ni-bearing Fe phases and a comparatively lower pH (Figs. 3 and 4). The Zn release from the CT-CPB was lower than that from CT, irrespective of binder fraction and the water saturation level. As unsaturated zones formed in the CT-CPB mixtures, the Zn release from the CE-FA mixtures increased, while the opposite occurred in the CE mixtures. A minor Zn release from CE446, compared with that from CE-FA446, could be due to a comparatively lower total content of Zn (Table 1), owing to the addition of a lower fraction of binder. Elements associated with the AEC fraction are often bound to carbonate phases that are sensitive to pH fluctuations. Carbonate phases formed under alkaline conditions are known to be thermodynamically unstable in the presence of atmospheric carbon dioxide (Benzaazoua et al. 2004). At the surface (with a lower water saturation level than the bulk) of CE446 and CE-FA446, carbonation resulting in dissolution of the cementitious phases may have occurred. In CE446, this yielded a reduction in Zn leaching, where most of the cementitious Zn phases on the surface dissolved, thereby forming more acid-tolerant species. A Zn decrease may have also resulted from the fact that the leachate/CPB contact area during a surface wash-controlled release (as in CE446) is lower than the area associated with a dissolution-like release (as in CE-FA446). In CE-FA446, more Zn is released at neutral pH than under acidic conditions (Fig. 3). That substantiates a Zn release from FA, which might contain Zn attached to calcium alumino-silicate glass that is more soluble under alkaline conditions than acidic, suggested by Lou et al. (Luo et al. 2011). By adding binders to CT, a significant fraction of Cu, Ni, and Zn might have been re-distributed from the water-soluble fraction to AEC phases in CE446 and CE-FA446. However, a large part of Zn, Cu, and Ni associated with the AEC fraction in CE446 and CE-FA446 could also originate from the binder themselves, as previously suggested in many studies (Pöykiö et al. 2011; Nurmesniemi et al. 2008; Świetlik et al. 2013). In these cases, maintaining high water saturation levels in the CT-CPB mixtures is essential to preventing the effects of ongoing pyrrhotite oxidation.

### Mobility of Ni, Cu, and Zn in CT and CT-CPB mixtures during accelerated weathering

Ni release from CT seemed to be correlated with the water-soluble Ni phases. This initially high release decreased sharply and diminished toward the end of the WCT, in contrast to the Zn release that was low during the WCT. The Cu release seemed to be correlated with the Fe-release, increased rapidly as the pH dropped below 4 (Fig. 5). The pH increased after the binders were added. Moreover, the results from the fractionation suggested that a large fraction of water-soluble phases was re-distributed into AEC phases (Fig. 1), which are sensitive to pH fluctuations (Dold 2003). In the CT-CPBs, the Cu, Ni, and Zn release exhibited similar trends and the maximum levels occurred with a sharp decrease (from 11 to 8) in pH until day 10. The release of Cu, Ni, and Zn decreased when the pH of the CT-CPB leachates stabilized at 8 (Fig. 5). This suggested that these elements are released, owing to desorption from Fe precipitates and/or the dissolution of cementitious phases. When acid was added to the CT-CPBs, the cumulative release of Ni, Cu, and Zn from the CT-CPB mixtures was substantially lower than that from the CT. This may have resulted from the fact that the pH at the endpoint (3.2) of the CT leachates was lower than that (pH: 4.5) of the CT-CPB leachates. The reaction kinetics of the WCTs differed from that of the TLT. In this case, the solubility of Cu, Ni, and Zn depended mainly on the pH and, hence, the elements were probably released via the dissolution/precipitation phases in the weathered CPB samples.

## Conclusions

Flooding of cemented paste backfill (CPB)-filled mine facilities is, commonly, a slow process where unsaturated zones may form within the CPB mass. Cyanidation tailings (CT) with elevated concentrations of Cu, Ni, and Zn and a pyrrhotite content of 1 wt% were managed by using CPB (CT-CPB). Two low strength (0.2 MPa) CPB mixtures were used, CE (tailings mixed with 1 wt% cement) and CE-FA (tailings mixed with 2 wt% of cement and 1 wt% of fly ash). Using a low strength CPB resulted in decreased leaching of Zn and Ni, regardless of water saturation level and binder proportion compared to that occurring in the CT. The establishment of unsaturated zones within the CT-CPBs leads to significant pyrrhotite oxidation, which resulted in acidity and, in turn, dissolution of the cementitious phases. At this stage, the mobility of Zn increased in tandem with pH in the CT-CPB mixtures. In CE mixtures, semi-acidic conditions established, Zn-release decreased whereas relatively stable Zn-species formed. In CE-FA mixtures, a dissolution of larger amounts of binders caused alkaline conditions to be established, and Zn release increased due to the solubility of fly ash-specific Zn species. A Ni release was low irrespective of binder proportions and unaffected by varying saturation levels. In CE, an establishment of unsaturated zones increased Cu release to be higher than that from CT. This increase resulted possibly from the formation of Cu phases that were less acid tolerant than those comprising the CT. The aforementioned results indicated that using a low strength CPB is not an environmentally friendly option for underground storage; otherwise, large amounts of Cu could be released.

## Appendix

**Table 4:** Results from the sequential extraction test (results expressed in mg/kg, *n* = 3, ± SD)

Ore		Water soluble	AEC	Fe(III) oxyhydroxides	Fe(III) oxides	Sulfides/residual	Total
Ca	mg/kg	160 ± 3	1427 ± 115	–	–	44,652 ± 2980	46,249 ± 3111
Fe	“ “	1.58 ± 0.39	1968 ± 23	3014 ± 83	2916 ± 57	97,606 ± 4689	105,506 ± 4693
Si	“ “	13 ± 1.1	57.5 ± 5.2	346 ± 17	643 ± 26	336,282 ± 1367	344,410 ± 9056
Cu	“ “	–	0.02 ± 0.01	0.64 ± 0.03	–	22 ± 0.7	23 ± 1.0
Ni	“ “	0.01 ± 0	5.1 ± 0.34	3.05 ± 0.11	0.65 ± 0.06	58 ± 1.4	67 ± 2.2
Zn	“ “	0.16 ± 0.17	2.37 ± 1.52	3.21 ± 1.55	1.37 ± 1.18	19 ± 0.2	51 ± 22
S	“ “	86.5 ± 0.5	0 ± 0	–	173 ± 21	10,084 ± 268	10,354 ± 304
CT							
Ca	mg/kg	3346 ± 56	227 ± 29	–	–	41,684 ± 1691	45,258 ± 1666
Fe	“ “	41.4 ± 2.56	353 ± 3	36,495 ± 202	5761 ± 303	79,122 ± 2046	121,772 ± 2656
Si	“ “	21.7 ± 0.7	28.1 ± 0.4	960 ± 4	673 ± 22	342,727 ± 9023	337,341 ± 1370
Cu	“ “	8.99 ± 0.69	0.82 ± 0.07	31.1 ± 0.8	–	54 ± 7.9	96 ± 8
Ni	“ “	36 ± 3	1.75 ± 0.42	3.7 ± 0.12	0.8 ± 0.08	4.5 ± 0.8	47 ± 2.7
Zn	“ “	9.36 ± 0.96	2.92 ± 2.56	6.48 ± 0.18	2.7 ± 1.76	30 ± 16	51 ± 22
S	“ “	5268 ± 141	1626 ± 44	1053 ± 13	807 ± 136	6312 ± 991	15,066 ± 998
CE-FA446							
Ca	mg/kg	2443 ± 132	11,915 ± 250	0 ± 0	39 ± 5.4	37,405 ± 2124	51,802 ± 1953
Fe	“ “	0.3 ± 0	2104 ± 78	15,624 ± 908	5795 ± 556	92,400 ± 3208	115,923 ± 2795
Si	“ “	46 ± 0.4	1453 ± 212	2206 ± 198	1271 ± 24	278,818 ± 8801	283,793 ± 8717
Cu	“ “	0.1 ± 0.0	28 ± 0.9	35 ± 2.1	0.3 ± 0.1	101 ± 3.9	165 ± 6.6
Ni	“ “	0 ± 0	12 ± 1.7	21 ± 1.2	1.9 ± 0.2	33 ± 2.4	67 ± 1.7
Zn	“ “	1.1 ± 0.6	15 ± 2.4	22 ± 1.7	5.3 ± 0.5	15 ± 0.7	58 ± 2.1
S	““	2082 ± 100	2221 ± 183	200 ± 22	83 ± 17	16,700 ± 755	21,287 ± 805
CE446							
Ca	mg/kg	7378 ± 112	707 ± 38	0 ± 0	21 ± 0.6	38,119 ± 1327	43,560 ± 3396
Fe	“ “	0.2 ± 0.1	458 ± 14	19,086 ± 96	3756 ± 482	90,300 ± 700	105,721 ± 13,356
Si	“ “	25 ± 0.7	212 ± 5.7	2075 ± 30	917 ± 72	263,856 ± 7142	265,977 ± 5412
Cu	“ “	0.0 ± 0.0	6.0 ± 1.4	35 ± 1.8	0.9 ± 0.2	107 ± 2.0	135 ± 22
Ni	“ “	0.8 ± 0.1	5.5 ± 0.3	30 ± 0.7	1.8 ± 0.2	30 ± 0.6	55 ± 21
Zn	“ “	4.8 ± 1.2	9.8 ± 1.2	25 ± 6.1	4.1 ± 1.4	14 ± 1.2	46 ± 29
S	“ “	6766 ± 91	228 ± 31	73 ± 1.9	36 ± 2.2	14,600 ± 700	19,370 ± 4188
